# Reflections on the Importance of Cost of Illness Analysis in Rare Diseases: A Proposal

**DOI:** 10.3390/ijerph18031101

**Published:** 2021-01-26

**Authors:** Patrizio Armeni, Marianna Cavazza, Entela Xoxi, Domenica Taruscio, Yllka Kodra

**Affiliations:** 1 Cergas (Centre for Research on Health and Social Care Management), SDA Bocconi School of Management, 20136 Milan, Italy; patrizio.armeni@unibocconi.it; 2 Independent Pharmacologist Scientific Advisor in Rare Disease Pharmaceuticals and Registries, 00184 Rome, Italy; entelaxoxi@gmail.com; 3 National Centre for Rare Diseases, Istituto Superiore di Sanità, 00162 Rome, Italy; domenica.taruscio@iss.it (D.T.); yllka.kodra@iss.it (Y.K.)

**Keywords:** rare diseases, social economic burden, cost of illness, health technology assessment

## Abstract

In the field of rare diseases (RDs), the evidence standard is often lower than that required by health technology assessment (HTA) and payer authorities. In this commentary, we propose that appropriate economic evaluation for rare disease treatments should be initially informed by cost-of-illness (COI) studies conducted using a societal perspective. Such an approach contributes to improving countries’ understanding of RDs in their entirety as societal and not merely clinical, or product-specific issues. In order to exemplify how the disease burden’s distribution has changed over the last fifteen years, key COI studies for Hemophilia, Fragile X Syndrome, Cystic Fibrosis, and Juvenile Idiopathic Arthritis are examined. Evidence shows that, besides methodological variability and cross-country differences, the disease burden’s share represented by direct costs generally grows over time as novel treatments become available. Hence, to support effective decision-making processes, it seems necessary to assess the re-allocation of the burden produced by new medicinal products, and this approach requires identifying cost drivers through COI studies with robust design and standardized methodology.

## 1. Introduction

In recent years, the peculiarities of evaluations and decisions surrounding the pricing and reimbursement of therapies for rare diseases (RDs) have been in the spotlight of academic debate and discussions among clinicians, health economists, and policymakers. The quality of scientific evidence is often lower than that required or expected by regulatory, Health Technology Assessment (HTA) bodies and payer authorities. In the case of RDs, indeed, it might be impossible to conduct large randomized clinical trials, and economic evaluation techniques may be unable to adequately reflect preferences of patients and society [1]. At the same time, treatments (very often orphan medicinal product, OMPs) for RDs are in most cases offered at a price deemed high by national authorities (payers and providers), especially if compared to the apparently modest clinical benefit characterizing a substantial number of OMPs. In other words, by using the standard set of methods and decisional approaches, RD treatments (RDTs) are likely to appear as low value for money. Consequently, many countries have adopted integrative policies to mitigate the influence of strictly evidence-based decision-making approaches (i.e., standard HTA approaches). A recent review focused on 12 European countries and specifically on RDTs, reported that the most common policies include the creation of national plans, and setting-up disease or drug-based registries [2]. In addition, the national healthcare payers have been increasingly looking into innovative reimbursement approaches and applying value-based pricing. In this regard, the use of Managed Entry Agreements (MEA) is recognized in order to manage the clinical and economic uncertainties [3]. These interventions favor early diagnosis through newborn screening, enable early patient access to new OMPs, particularly those targeting an unmet medical need or addressing a major public health interest (e.g., compassionate use and new regulatory pathways like PRIME (PRIority MEdiciness) scheme or Adaptive pathway) and following dedicated HTA approaches. All these policies are likely to facilitate access to new therapies for RDs. However, they exogenously overcome the perception of “poor value for money” without providing credible reasons for why such therapies could also prove to be a reasonable investment from a health-economic standpoint. As a matter of fact, besides the arguments reported by Simoens [4], one of the main challenges for economic evaluation of therapies for RDs is the little number of active comparators, that is, most of these therapies are launched without any previous treatment indicated for the same condition. To this is added the use of surrogate endpoints not yet validated or currently being validated, which complicates the correlation of a possibly statistically significant endpoint, but not a clinically relevant outcome. In this perspective, economic evaluation would record in many cases a pure increase in the cost of therapies.

In this commentary paper, we would like to show how Cost of Illness (COI) studies (also known as Burden of Disease) conducted using a societal perspective can effectively inform appropriate pharmacoeconomic analysis for OMPs or RDTs. This approach is able to show the distribution of the disease burden (i.e., the consequence of health problems on a given population in terms of mortality, morbidity, quality of life, or financial costs, etc.) over the different actors impacted by the condition, i.e., the patient, her/his caregiver(s), the healthcare sector, pharmaceutical industry, other public institutions providing financial or organizational support, employers, etc.

COI should represent the status quo for budget impact analysis, extended to society perspective when new treatments are launched in the market. Specifically, this type of study shows a) how much the overall financial and economic burden is impacted by the new treatment and, b) to what extent does the new treatment reallocate that burden over the different actors (e.g., displacing it from patients and caregivers to the healthcare system).

Three arguments sustain our observation. First, when no indicated treatment is available, it is likely that the direct healthcare costs are low and, therefore, the launch of a new (and typically high-priced) medicinal-product (MP) heavily impacts the financial engagement of healthcare payers on each patient. In this light, evaluations conducted using the payer’s perspective will be easily biased towards the high delta cost, while the consideration of the overall burden would help to evaluate the extent of a shift in the burden from patients and other societal actors (e.g., social security systems, families or employers, etc.) to payers. Second, consideration of the overall burden of disease allows HTA agencies and payers to identify the real economic impact of a new high-priced MP on the healthcare system (thus anticipating the information of a budget impact analysis (BIA), but from the broader societal perspective), which in the case of RDs is generally modest [5]. Third, such an approach contributes to countries’ understanding of RDs in their entirety as societal and not merely clinical or product-specific issues, thus reframing the concept of value for money as a reduction of the overall burden of disease, irrespective of what actor bears the greatest share. In the examples provided below, even if not generated as systematic evaluations by policy-makers, the evolution of the burden’s allocation over time (when ad hoc new treatments become available) can clearly be seen.

## 2. Methods

**Figure 1:**
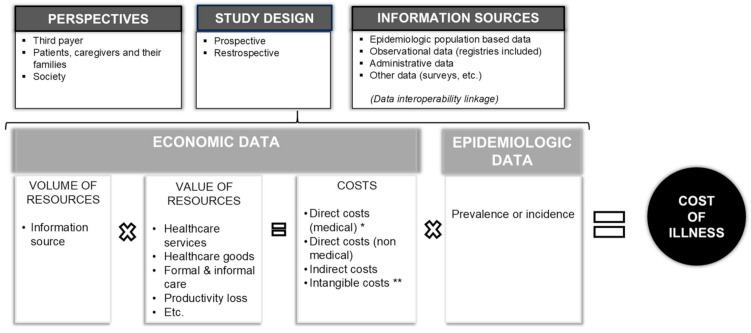
The key factors in determining the cost of illness. * For drugs: Eventual managed entry agreements (label use) or early access use. ** For correctness, let us also mention intangible costs, even if we did not mention them in this commentary because they are not easily estimated and used in the scientific literature.

In order to exemplify how the burden’s distribution has changed over the last fifteen years, we identified four examples of RDs to support our observation: Hemophilia, Fragile X syndrome, Cystic Fibrosis, and Juvenile Idiopathic Arthritis, representing a broad spectrum of RDs. In selecting these diseases, we take advantage of previous studies carried out by the Social Economic Burden and Health-Related Quality of Life in Patients with Rare Diseases in Europe Project—BURQOL-RD. Therefore, we applied the BURQOL project’s same criteria (genetic origin, age at onset during adulthood or childhood, physical impairment and/or mental impairment, availability of effective therapies) in identifying effective examples of RDs for our analysis [6]. Hence, key articles focused on the socioeconomic evaluation of RDs, in particular applying a societal perspective, were selected. Figure 1 shows the structure of a COI study and how this is not a comparative approach, as Cost Effective Analysis (CEA) and Cost Utility Analysis (CUA) are, and it adopts a societal perspective, as CEA and CUA usually do not. In turn, this wide perspective of society allows us to effectively identify which actors hold in the greater extent of the disease burden, but it also requires a wider range of data than CEA and CUA.

Even if our goal is not a systematic review of the literature or a meta-analysis, we considered those items of the PRISMA checklist concerning data items and their source, collection, measurement, and processing in all studies published during the period 2004–2019 focused on socioeconomic evaluation of RD examples, applying a societal perspective of cost of illness methods [7]. Additional inclusion criteria were used for study selection concerning: (i) Detailed description of the data source; (ii) reported data collection procedures; (iii) use of appropriate measures of costs; (iv) methods for cost data processing described in adequate detail; (v) provided estimates of indirect or direct costs for each disease. Lastly, we excluded reviews of existing economic studies relating to specific diseases and studies with partial estimation of costs.

The research was conducted searching the PubMed database and using the following terms: (i) Economic burden and (ii) costs in combination with the names of the four selected RDs.

Specifically, items considered for description of each study were:

- name of RD;
- publication year;
- country;
- study population (target and size);
- perspective (i.e., societal, third payer, patients, and families);
- study methodology;
- data source;
- annual average direct health care cost per patient (including all types of healthcare costs directly related to the studied disease from diagnosis and treatment to continuing care and rehabilitation);
- annual average direct formal non-healthcare cost per patient (including costs of transportation);
- annual average direct informal non-healthcare cost per patient (including informal care by non-professional caregivers such as family members or friends, etc.);
- annual average indirect cost per patient (productivity losses);
- total annual average cost per patient;
- costs were reported in terms of absolute values and percentage distribution referring to the total costs.

The search identified 49 citations (12 papers for Hemophilia; 7 papers for X Fragile Syndrome; 25 papers for Cystic Fibrosis; and 5 papers for Juvenile Idiopathic Arthritis). All articles were screened by title and abstracts to identify those that fit the above mentioned inclusion criteria. After excluding 34 papers that did not meet inclusion criteria, a total of 15 papers were included for full text analysis. The papers were assessed independently for inclusion by two authors.

Table 1 provides information regarding the main study characteristics and types of costs included, such as the direct health costs, direct non-health care costs (formal and informal), and indirect costs. While Table 2 shows the cost items most frequently considered in the analyzed COI studies.

## 3. Results

An overview of the COI approach and the cost items most frequently considered in the analyzed COI studies is provided by Table 2, and a more detailed description of results for each sampled RD follows.

Hemophilia A is a rare, X-linked bleeding disorder that affects approximately 1 of every 5000 to 10,000 live-born males [23]. Hemophilia B is much less common than A, with an incidence of approximately 1 in 25,000 births [24]. A definitive diagnosis of hemophilia A or B is typically made based on an established family history and/or patients’ presentation of a bleeding event that has been confirmed by laboratory tests to be the result of coagulation factor deficiency. The clinical presentations of the two conditions are indistinguishable, and the bleeding tendencies associated with each disorder tend to correlate directly with plasma concentrations of factor VIII (FVIII) and factor IX (FIX), respectively. Recurrent bleeding into the joint and muscles leads to irreversible bone and cartilage damage, culminating in disabling hemophilic arthropathy. Treatment for hemophilia A or B involves routine administration of exogenous coagulation factors to replace the missing/deficient endogenous FVIII or FIX, respectively [25]: This treatment implementation has changed continuously through the years in terms of products used (i.e., by-pass agents for patients with inhibitors, recombinant and monoclonal antibody drugs replacing Factor VIII) and administration method (on demand and prophylaxis administration) [25]. The overall cost of treatment remains high, particularly among patients with more severe forms of hemophilia [24]. Clotting factors have been estimated to account for approximately 90% of the direct health care costs for hemophilia management, and costs varied based on the treatment approach, patient characteristics in terms of age or comorbidities, and disease severity [26,27].

The COI analyses focusing on hemophilia covered several European countries, including two cross-countries studies [8,9], while one considers the hemophilia burden in the USA [10]. Due to the increasing availability of ad hoc treatments [25], direct costs are the main cost driver and range from 77% to 97% of total costs. In some cases, the included cost items consider only those drugs and healthcare services strictly related to the hemophilia treatment [11]; in other cases, direct cost items address a wider range of healthcare services providing the perspective of the whole process of care (i.e., primary care visits or dental care) [15]. Direct formal non-healthcare costs refer to patient transport expenses, and one study also considers informal care [8,10]. Last, the human capital approach seems to prevail in these instances, as sick leave and absenteeism along with early retirement are considered.

*Fragile X Syndrome* (FXS) is a chromosome X-linked genetic condition that leads to intellectual disability, learning and behavioral challenges, and physical characteristics [28]. This neuro-developmental disorder occurs in both sexes; about 1.4 per 10,000 males and 0.9 per 10,000 females have FXS, and European Union (EU) prevalence is estimated to be 32 per 100,000 inhabitants [29]. FXS is on average diagnosed around age 3, when cognitive and developmental disabilities become apparent. The severe comorbid profile of individuals with FXS contributes to high healthcare costs and utilization of medical specialist visits, tests or procedures, and pharmacotherapy management. The burden of FXS is not only on the patient; similar to patients with other developmental disabilities (DD), family caregivers of patients with FXS face multiple challenges. The caregiver burden from FXS is mostly related to managing behavioral symptoms associated with the disease [30]. Studies have found that families affected by FXS report an increased financial burden, and over 60% stated that they had to change work hours or quit jobs because of having a child with FXS [31]. Multidisciplinary treatment, including speech and language therapy, occupational therapy, physical therapy, special education, behavioral interventions, and genetic counselling, are needed for patients affected with FXS.

Unlike hemophilia, few studies analyze the social and economic burden of the Fragile X Syndrome from the societal perspective. To date, FXS is generally treated with selective serotonin reuptake inhibitors along with several long-term, non-medical interventions, and no ad hoc, specific treatment has been made available yet. Table 1 provides two instances of COI studies: The first includes not only direct healthcare costs and indirect costs, but also direct formal and informal non-healthcare costs, showing how these two items are relevant to this type of disease (i.e., 40% and 48%, respectively). Moreover, indirect costs show the impact of informal care on parents’ working condition [13]. The second study adopts a third payer perspective and then focuses just on healthcare services and goods consumed by FXS patients [14].

*Cystic fibrosis* (CF) is a rare genetic (autosomal recessive), multi-systemic, fatal disease that starts at birth and progresses over time to lung failure. CF is caused by a reduced quantity and/or impaired function of the CF transmembrane conductance regulator (CFTR) protein due to mutations in the CFTR gene. CF is mainly characterized by progressive lung function decline. This decline in lung function increases the risk of pulmonary exacerbations [32], which further exacerbates lung function decline and increases the risk of death [33,34]. Moreover, patients may experience extensive damage in multiple organs before presenting with symptoms or a decline in ppFEV1. Clinical presentation and progression vary with genotype. CF substantially impacts patients’ health-related quality of life and results in early death, regardless of genotype.

Prenatal diagnostics, newborn screening, and new treatment algorithms are changing the incidence and the prevalence of the disease. CF has an estimated prevalence of approximately 0.737 per 10,000 inhabitants in Europe [35]. Although progress in early diagnosis and new therapeutic strategies are also substantially improving the prognosis of CF and, currently, average life expectancy of patients exceeds 40 years. Only recently (from 2012), novel therapies (CTFR modulators) [15] that directly correct errors caused by mutations of the CFTR gene are becoming available in the EU for patients with certain mutations in CFTR protein.

Existing treatments for CF can be broadly classified in two groups: (1) Therapies managing the symptoms, complications, and comorbidities of the disease (e.g., antibiotics, mucolytics, pancreatic enzyme replacement therapy) and (2) CFTR modulators (i.e., correctors and potentiators), which target the underlying cause of the disease. Regarding the latter, four medicinal products (with orphan designation) have been approved by the European Commission [15]. Concomitant administration of these two groups is recommended to maintain and improve lung function, reduce the risk of infections and exacerbations, and improve quality of life. CFTR modulators, targeted at the underlying cause of CF rather than symptoms, have improved survival with short- and long-term improvements in clinical outcomes, but they have also increased costs causing difficulties in their availability for many countries. Therefore, given changing life expectancy and high cost of novel drugs, resources dedicated to CF care are very likely to have substantially increased in recent years [36,37,38].

The socioeconomic literature on CF provides effective instances of the treatments’ impact on disease cost. The studies described in Table 1 [15,16,17,18] show how much treatment costs may impact on the balance of a disease with an ad hoc therapy, and how the inclusion of a wide perspective of indirect costs [15,17,18] and informal care [17] can cast a different light on high direct healthcare costs. For instance, the direct medical costs in Germany [33] are mainly driven by high drug prices since the introduction of very expensive CF mutation-specific drugs in 2012; even if, as the authors also reported, costs increase with disease severity and related complications. The two German studies [15,16] have different evaluation perspectives: Heimeshoff et al. adopt a societal perspective [15], noting that the relevance of indirect costs is likely to increase in the future as life expectancy of CF patients increases. While Frey et al. assume a payer perspective [16] and contend that CF has a constant and wide-ranging economic impact on payers, with considerable differences in the distribution of costs and service utilization between younger and older patients, as well as mild vs. severe patients. Hence, they remind us how pharmaceutical expenses will increase in the future as causative treatment gains importance.

*Juvenile Idiopathic Arthritis* (JIA) is currently grouped in multiple categories, some of which have counterparts in the more frequent adult diseases of rheumatoid arthritis, axial spondyloarthritis, and psoriatic arthritis, though with considerable differences in phenotype at different ages. The etiology of JIA is unknown and the pathogenesis unclear, but it is likely multifactorial [39]. JIA is the most common chronic rheumatic disease in childhood, with an incidence of 10–19/100,000 children below the age of 16 years, and it is also one of the major causes of acquired disability and impairment of quality of life in childhood [29]. Early and aggressive control of arthritis is essential to prevent long-term disability [40]. JIA has an overall prevalence estimate in the EU of 16-150 cases per 100,000 inhabitants [41]. Without appropriate treatment, JIA may result in devastating consequences. Children may experience permanent disability from joint destruction, growth deformities, or blindness. Drug therapy covers a broad spectrum of medicines, e.g., non-steroidal anti-inflammatory agents, intra-articular corticosteroid injections, disease-modifying anti-rheumatic drugs, anti-interleukin therapy, and biologicals. The choice of medication depends on the subtype of JIA. However, treatment still needs to be developed. The hope is that recent changes in treatment approaches will result in marked improvement in long-term functional outcomes of patients with JIA.

JIA literature considers a wide range of healthcare services, aids, and goods representing direct healthcare cost items. Direct non-healthcare costs include formal care in two papers [19,21], while only one considers also informal care [21]; moreover, the included items effectively represent the burden on families in terms of transport, home alterations and professional care, etc. A good example of the extent of variability in this type of analysis is the indirect costs estimation by Minden et al. in 2004 and 2009 in Germany [19,22]. Indeed, Minden et al. [22] in 2004 adopt a human capital approach including both loss of productivity in terms of sick leave and work disability, implying an entitlement to a social security pension; while Minden et al. [19] in 2009 define loss of productivity as lost working days by parents.

## 4. Discussion

RDs have been an increasing area of focus, as three effects have converged in recent years: (i) Continuing innovation stemming from the genomic revolution, (ii) regulatory and financial incentives for RDTs, and (iii) the increasingly mobilized, coordinated, and sophisticated patient community. The cost of RDs, and by corollary the opportunity for savings, continues to increase.

We posit that COI provides critical information needed by policymakers when dealing with new RDTs. To show the type of evidence made available so far, we analyzed examples of COI papers published in the last decade on four selected RDs. These results showed significant heterogeneity in identifying all cost items associated with the selected RDs, consistent with results from Angelis et al. [42], suggesting the following reflections. First, the existence of a pharmaceutical treatment is positively correlated with the availability of cost analyses; second, indirect costs and direct non-healthcare costs account for a relevant portion of total costs when there is no specific indicated therapy (e.g., FXS), but only symptom treatment.

There are several characteristics related to COI studies requiring attention (Table 3). First, it is the need of reliable datasets addressing all information related to all types of costs of rare or ultra-rare conditions, often unavailable or not well-structured, which constitutes a problem for drug-developers, regulators, and particularly for HTA bodies and payers. Second, the four disease examples addressed in this paper demonstrate the high variability of costs depending on different perspectives. This variability, strongly linked with disease characteristics, is correlated with the level of innovation in treatments for the specific rare therapeutic area. Innovation can modify the course of the disease and in some cases—when there is an added clinical value—change the natural history of the disease. Hence, it is almost mandatory to trace this flow of transformation over time and to monitor both clinical aspects and associated costs. Third, COI studies are still relatively scarce compared with economic evaluations in general and epidemiological studies: The reasons for this scarcity might be in part the difficulty of linking diagnoses and clinical outcomes to resource utilization and in identifying costs outside the healthcare system.

However, from a positive perspective, the category of COI covers several key aspects ranging from the incidence or prevalence of disease to its effect on longevity, morbidity, health status, and quality of life, and also considers related financial aspects, including direct and indirect expenditures resulting from premature death, disability, or injury due to corresponding disease and/or its comorbidities [43]. Moreover, this wide range of perspectives provides detailed indications regarding which actor is bearing the social economic and care burden, and where it is shifting as a new treatment is introduced.

Therefore, COI studies are necessary for the decision-making process at national and local levels: From a payer perspective, it is essential that the COI be a structuring part of the national dossiers for drug price negotiating. Alongside the assessment of unmet medical needs (mostly maximum level for the rare condition), the added clinical value of a new treatment (the most challenging criteria) and the robustness of clinical trials (often low or very-low), COI studies would help to better complete the overall rare disease picture. This would also help the payer or decision maker identify cost areas (not necessarily only related to the RDs) and additionally to start planning any shifts of his pharmaceutical budget from one area to another more promising one (in terms of clinical benefit) or priority (public health needs). In particular for RDs, this approach would help not only in the case of treatments approved by regulatory authority (label indication), but also to evaluate an eventual early access program planned to be used in an RD context (e.g., Italian 648/96 Law or French ATU procedures) [44,45].

It should be highlighted that the burden of disease (or COI) is an element of uncertainty, and, like all the other uncertainties that characterize a new product upon introduction in a specific therapeutic area, COI should also be managed [46]. For several years now, various payers have been applying the MEAs [3] as a set of tools aimed at facilitating access to new medicines in order to manage clinical (performance-based risk-sharing agreement) and financial (budget impact) uncertainties (See Figure 1). Patient registries are also used to verify appropriateness and implement Managed Entry Agreements [47,48].

In the last years, some initiatives have been developed to deal with economic challenges of rare diseases: BURQOL-RD [49,50], a multinational study, recently evaluated the burden of ten rare diseases in Europe, using a prevalence-based method with a bottom-up approach to quantify resources from a societal perspective, which is the mostly used methodology for these studies in rare diseases. Several programs at the European level (i.e., EUnetHTA [51], FP7 Advance HTA [52], IMPACT HTA [53]) are addressing HTA use and dissemination even in the field of RDs, improving and strengthening the methodological tools and practices.

## 5. Conclusions

We believe that COI studies are useful to inform policy-makers in evaluating the full impact of proposed new treatments for RDs, but methodological heterogeneity makes available studies difficult to compare. To effectively support decision-making processes, it is necessary to assess re-allocation of the burden of disease produced by new treatments, requiring identification of cost drivers through COI studies with robust design and standardized methodology.

## Figures and Tables

**Table 1:** Samples of cost of illness studies concerning the selected rare diseases.

Name of Rare Diseases	Authors and Pubblication Year	Country	Study Population (Target and Size)	Perspective	Study Methodology	Data Sources	Annual Average Direct Formal Health Care Cost per Patient	Annual Average Direct Formal non Healthcare Cost per Patient	Annual Average Direct Informal non Healthcare Cost per Patient	Annual Average Indirect Cost per Patient	Total Annual Average Cost per Patient	Reference Number
Haemofilia	Cavazza M et al., 2016	Bulgaria, France, Hungaria, Italy, Germany, Spain, Sweden, UK	339 adult and child patients with hemophilia A and B	Societal perspective	A cross-sectional study. Cost analysis based on a bottom-up approach	Patients’ survey	Items: Rehabilitation, medical tests and examinations, visits to health professionals and home medical care) emergency visits; drugs, healthcare transport, and health materials. Value: €44,842.37 (78%)	Items: Professional care, social services, and non-healthacare transport. Value: €1896.37 (3%)	Items: Informal care. Value: €3119.43 (5%)	Items: Early retirement, sick leave. Value: €7518.33 (13%)	Value: €57,376.51 (100%)	[8]
	O’Hara J et al., 2017	France, Germany, Italy, Spain and UK.	551 adult patients with severe hemophilia A and B without inhibitors	Societal perspective	A retrospective, non-interventional study	Clinicians and patients’ survey	Items: Ambulatory (Haematologist visit, nurse per visit, other specialist visits, blood tests, other tests, drugs) and hospitalization (target joint procedure, bleed event: Ward stay (per day)), Bleed event: ICU stay (per day) costs. Value for clotting factor replacement therapy (CFRT)): €189,285.00 (95%). Value for other medical costs: €4181.00 (2%)			Items: Wage (patient/caregiver), petrol (per mile). ** Value: €6075.00 (3%)	Value: €199,541.00 (100%)	[9]
	Chen CX et al. 2017	USA	112 patients children and 50 adults with hemophilia B	Societal perspective	Prospective study with longitudinal cohort data	Clinicians and patients’ survey, and administrative data	Items: Inpatient services (all-cause hospitalizations, emergency room (ER) visits), outpatient services (comprehensive, nursing, clinician, physicaltherapist, and socialwork/psychology), laboratory tests, and outpatient procedures), and medication costs (including clotting or bypass treatments). Value for all patients: $ 133,894.00 (95%) Value for mild patients: $ 51,435.00 (92%) Value for severe patients: $ 190,312.00 (87%)			Items: Lost wages due to days of work absenteeism among those employed and unpaid hemophilia-related caregiver time reported; hemophilia-related part-time employment or unemployment reported. Value for all patient: $ 6346.00 (5%) Value for mild patients: 4416.00 $ (8%) Value for severe patients 8421 $ (13%)	Value for all patients: $ 140,240.00 (100%) Value for mild patients: $ 85,852.00 (100%)Value for severe patients: $ 198,733.00 (100%)	[10]
	Café A et al., 2019	Portugal	127 adult and child patients with hempphilia A and B	Societal perspective	A mix of retrospective and probabilistic model	Experts panel and patients’ survey, administrative data and national literature data	Items: Hemophilia related hospitalization; outpatient care (physicians, nurses, physiotherapists, etc., visits, laboratory and imaging exams, concomitant medications (for pain), hemophilia treatment. Value: €50,255.47 (88%)	Items:Transportation to medical appointments. Value: €3692.53 (6.5%)		Items: Unemployment rate, labor absenteeism; early retirement. Value: €2927.00 (5.5%)	Value: €56,875.00 (100%)	[11]
	Henrard S et al., 2017	Belgium	A simulation of new-born males with hemophilia A and B in 2011 and male births in 2011 in Belgium with a hemophilia A and B incidence from 1/5500 to 1/4500 new-born males in Belgium	Societal perspective	Prospective study	Administrative data and friction-cost method	Items: Hemophilia medications, hospitalization, general practitioner (GP), specialist, physiotherapist, and dentist. Value: €180,517.11 (97%)	Items:Transport costs to and from the doctor’s office and hospital. Value:€3692.530 (2%)		Items: Absence from work due to these appointments and hospitalisations; absence from work due to invalidity or premature death. Value: €1880.50 (1%)	Lifetime value: €97.4 million (95% CrI: €47.1–158.1 million)	[12]
Fragile X Syndrome	Chevreul K et al., 2016	France	147 adult and child patients	Societal	Cross sectional study	Patients recruited through the French FXS patient associations	Items: Rehabilitation, medical tests and examinations, visits to health professionals and home medical care) emergency visits; drugs, healthcare transport, and health materials. Value: €2687.00 (10%)	Items: Professional care, social services, and non-healthacre transport. Value: €10,511.00 (40%)	Items: Informal care. Value: €12,586.00 (48%)	Items: Early retirement, sick leave. Value only for adult patients: €31,240.00	Value: €25,784.00 (100%) *	[13]
	Sacco Pet et al., 2013	USA	721 patients with Medicaid (all age)	Third payer perspective	Retrospective observation cohort study	Patients recruited from Medicaid databases	Items: Emergency department visits, hospitalizations, outpatient visits, medical procedures. Value range: $ 4548.00–$ 9702.00		Items: Informal careValue: €3119.43			[14]
Cystic Fibrosis	Heimeshoff M et al., 2012	Germany	158 adult and child patients with severe CF	Societal perspective	Prospective study	Administrative data, register of CF pstients, clinicians, and healthcare professional survey	Items: Drugs, laboratory tests, staff cost per patient, and centre’s overhead. Value: €38,869.00 (93.7%)	Items: Transport. Value: €10,800 (0.3%)		Items: Early retirement a/o disability pensions provided by the social insurance system. Value: €2492.00 (6%)	Value: €41,468.00 (100%)	[15]
	Frey S et al., 2019	Germany	2241 patients with mild, moderate, and severe CF	Third payer perspective	Retrospective observation cohort study	Administrative claims data	Items: Outpatient treatment, drugs, care by non-physicians (e.g., physiotherapy), devices and medical equipment, inpatient treatments, rehabilitation and nursing care (at home). Value for patients with mild CF: €8920.00 (99.05%) Value for patient with moderate CF: €50,121.00 (99.5%) Value for patient with severe CF: €95,768.00 (99%)	Items: Services (e.g., transportation). Value for all patient with mild CF: €87.00 (0.95%) Value for patient with moderate CF: €269.00 (0.5%) Value for patient with severe CF: €994.00 (1%)			Value for patients with mild CF: €17,551.00 (100%) Value for patients with moderate CF: €50,390.00 Value for patients with severe CF: €96,762.00	[16]
	Chevreul K et al., 2015	France	240 adult and child patients	Societal perspective	Retrospective cross-sectional study	Patients’ survey	Items: Rehabilitation, medical tests and examinations, visits to health professionals, and home medical care) emergency visits; drugs, healthcare transport, and health materials. Value: €16,851.00 (46%)	Items: Professional care, social services, and non-healthacre transport. Value: €4512.00 (12%)	Items: Informal care. Value: €4827.00 (13%)	Items: Early retirement, sick leave. Value: €10,408.00 (28%)	Value: €36,598.00 (100%)	[17]
	Kopciuch D et al., 2017	Poland	46 adult patients	Societal perspective	Retrospective study	Patients’ survey and administrative data	Direct healthcare costs: Hospitalization, outpatient visits, pharmacotherapy, diagnostic tests. Value: €13,740.33 (70%),	Items: Transportation. Value: €57.80 (0.3%)		Items: Presenteeism. Value: €5782.94 (29.7%)	Value: €19,581.08 (100%)	[18]
Juvenile IdiopathicArthritis	Minden K et al., 2009	Germany	369 child patients	Societal perspective and patient’s perspective	An incidence based, retrospective study	Patients’ survey, medical records, and administrative data	Items: Pediatric rheumatology service use, ophthalmologist service use, Other JIA-related physician service use, non-physician service use, day-surgery, medication, devices and aids, acute hospital facilities, surgery, rehabilitation, comprehensive alternative (non-prescription) medicine. Value: €4172.00 (89%)	Items: Transportation, extra telephone, home alterations, domestic help and care. Value: €223.00 (5%)		Items: Loss of productivity. Value: €270.00 (6%)	Value: €4663.00 (100%)	[19]
	Yucel IK et al., 2012	Turkey	100 child patients	Societal perspective	A cross-sectional study	Patients and caregivers surveys	Items: Outpatient visits, biochemical tests, radiologicaltests, physiotherapy, hospitalization fees, surgery, drugs, devices, physiotherapy. Value:€3725.00 (94%)	Items: Transportation, lodging expenses. Value: €188.00 (5%).		Items: Work days lost among parents. Value: €81.00 (2%)	Value: €3994.00 (100%)	[20]
	Angelis AP et al., 2016	UK	23 child and adult patients	Societal perspective	A cross-sectional study	Patients and caregivers surveys	Items: Medication, tests, outpatient and primary health care visits, acute hospitalization, devices, healthcare transportation. Value: €14,508.00 (46%)	Items: Professional carer, non-healthcare transportation. Value: €722.00 (2%)	Items: Informal care. Value: €7621.00 (24%)	Items: Productivity loss, early retirement, and sick leave. Value: €8715.00 (28%)	Value: €31,546.00 (100%)	[21]
	Minden K. et al., 2004	Germany	215 child patients	Societal perspective and patient’s perspective	An incidence based, retrospective study	Patients’ survey, medical records, and administrative data	Items: Inpatient care (acute hospital facilities, surgery, non-acute hospital facilities (rehabilitation)); outpatient care (JIA-related rheumatology service use; other JIA-related physician service use; non-physician service use; surgery; medication; devices and aids. Value: €1821.00 (52%)		Items: Patient expenditures. (3 months) Value: €73.00 (2%)	Items: Loss of productivity. Value: €1571.00 (45%)	Value €3465.00 (100%)	[22]

* The total value does not include indirect costs as they refer only to adults. ** Even if petrol, related to transportation costs, is a direct formal non-healthcare cost, it was not possible to extrapolate this specific item from indirect costs.

**Table 2:** Summary of cost items considered by the samples of cost of illness studies.

		Hemophilia					Fragile X Syndrome		Cystic Fibrosis				Juvenile IdiopathicArthritis			
		Cavazza et al. [8]	O’Hara et al. [9]	Chen et al. [10]	Caféet al. [11]	Henrard et al. [12]	Chevreul et al. [13]	Sacco et al. [14]	Heimeshoff et al. [15]	Frey et al. [16]	Chevreul et al. [17]	Kopciuchet al. [18]	Mindenet al. [19]	Yucel et al. [20]	Angelis et al. [21]	Minden et al. [22]
Direct formal healthcare cost	Medical tests and exams	X	X	X	X	X	X	X	X	X	X	X	X	X	X	X
	Visits	X	X	X	X	X	X	X	X	X	X	X	X	X	X	X
	Hospitalization	X	X	X	X	X	X	X		X		X	X	X	X	X
	Rehabilitation	X		X	X	X	X			X	X		X	X		X
	ER access	X		X			X	X			X					
	Home healthcare						X			X	X					
	Healthcare transportation	X		X			X				X				X	
	Drugs and healthcare materials	X		X	X	X	X		X	X	X	X	X	X	X	X
Direct formal non-healthcare cost	Professional care	X					X				X		X		X	
	Home alterations												X			
	Social services	X					X				X					
	Non healthcare transportation	X			X	X	X		X	X	X	X	X	X		
	Lodging expenses													X		X
Direct informal non-healthcare costs	Informal care	X					X	X			X				X	
Indirect costs	Sick leave	X				X	X				X		X	X	X	X
	Abstenteism			X	X							X	X	X		X
	Unemployment		X	X	X											
	Early retirement	X			X	X	X		X		X				X	

X indicates which cost items (1st and 2nd left columns) are included in the COI estimation provided by analyzed papers (top line).

**Table 3:** “Pros” and “cons” of cost of illness (COI) analyses.

PROs	A COI analysis will help decision makers gain information on the current and/or prospective economic burden of a disease.
	If a societal perspective is adopted, the COI analysis will allow for the identification of those societal actors bearing most of the burden (which are often excluded or neglected by other economic evaluation methods).
	Over time, and with the availability of new therapies, updating the COI analysis will offer the opportunity to assess how much the burden has shifted from patients/caregivers to other actors (e.g., a third party payer).
CONs	COI is not a comparative analysis, so it should not be used to assess the opportunity of introducing a new therapy. However, when the economic burden of a disease is heavily concentrated on patients, caregivers, and society, with a small amount born by the healthcare system, this could be a signal that a (new) therapy is needed.
	Data sources are often scarce or outdated. This is a major problem with the use of COI: The more epidemiological data are sound and up to date, the more COI will be informative.
	COI describes the economic burden at a specific point in time: It needs frequent updates to keep its informative value. This requires the availability of human and economic resources that are generally not invested by national authorities.

## Data Availability

Not applicable.
